# The Transcriptional Regulator MucR, but Not Its Controlled Acid-Activated Chaperone HdeA, Is Essential for Virulence and Modulates Surface Architecture and Properties in *Brucella ovis* PA

**DOI:** 10.3389/fvets.2021.814752

**Published:** 2022-01-31

**Authors:** Beatriz Tartilán-Choya, Rebeca S. Sidhu-Muñoz, Nieves Vizcaíno

**Affiliations:** ^1^Departamento de Microbiología y Genética, Universidad de Salamanca, Salamanca, Spain; ^2^Instituto de Investigación Biomédica de Salamanca, Salamanca, Spain

**Keywords:** *Brucella ovis*, MucR, HdeA, virulence, Omp25d, transcriptional regulation, acid stress, deletion mutant

## Abstract

*Brucella ovis* is a non-zoonotic bacterium causing contagious epididymitis and other genital lesions in rams and responsible for significant economic losses in sheep-breeding areas. It is a naturally rough (without O-chains in the lipopolysaccharide) *Brucella* species whose virulence mechanisms have been less explored than those of zoonotic smooth brucellae (bearing O-chains that mask other outer membrane molecules). Considering the rough nature of *Brucella ovis*, the influence of surface components other than O-chains on its biological properties may be greater than in smooth *Brucella* species. Here we describe the construction and characterization of the *mucR* deletion mutant of virulent *B. ovis* PA, which is defective in a transcriptional regulator, affecting surface properties and virulence in smooth brucellae. This mutant showed increased amounts of three proteins identified as HdeA (acid-activated chaperone), Omp25d (outer membrane protein undetectable in the parental strain), and BOV_A0299 (hypothetical protein of unknown function). This observation correlated with the enhanced transcription of the corresponding genes and constitutes the first report on this type of proteome alteration in *Brucella* Δ*mucR* mutants. The upstream regions of the three genes contained AT rich domains with T-A steps described as binding sites for MucR in the *Brucella abortus* 2308 *babR* promoter (gene also upregulated in *B. ovis* Δ*mucR*), which suggests that *hdeA, omp25d*, and *BOV_A0299* expression could be repressed by MucR through a direct binding to their promoter regions. Relative quantification of transcripts of several other genes selected according to the transcriptome of smooth brucellae Δ*mucR* mutants revealed not only similarities but also relevant differences among strains, such as those detected in flagellar and *virB* genes. Periplasmic HdeA has been related to the resistance of *B. abortus* to acidic pH, conditions encountered by *Brucella* inside phagocytes, but the deletion of *hdeA* in *B. ovis* PA and the Δ*mucR* mutant did not modify any of the evaluated properties of these strains. The *B. ovis* PA Δ*mucR* and Δ*mucR*Δ*hdeA* mutants had defective *in vitro* growth and altered surface properties and architecture, exemplified by detectable amounts of Omp25d. Moreover, they showed virulence attenuation but established persistent splenic infection in mice, which encourages their evaluation as specifical attenuated vaccines against *B. ovis*.

## Introduction

The genus *Brucella* (https://lpsn.dsmz.de/genus/brucella) includes facultative intracellular Gram-negative bacterial pathogens that differ, among other phenotypic and genotypic characteristics, in pathogenicity and host preference (1, 2). Depending on the *Brucella* species, a variety of terrestrial and marine mammal species can be infected, but amphibians, reptiles, and fish have also been reported as hosts for the atypical *Brucella* strains described in the recent years (1, 2). Ovine brucellosis is mainly caused by *Brucella melitensis* and *Brucella ovis*, species that share high levels of homology at the DNA level (3) but exhibit relevant differences regarding pathogenicity. Thus, *B. melitensis* induces abortion in the natural host, is the most relevant zoonotic *Brucella* species (4), and is defined as smooth because it bears O-polysaccharide chains in the lipopolysaccharide (LPS), which are required for full virulence (5, 6). On the contrary, *B. ovis* is rough (lacks O-chains in the LPS), has never been reported as a human pathogen, and rarely induces abortion in sheep. However, *B. ovis* causes contagious epididymitis and other genital lesions in rams that originate important economic losses worldwide (7, 8) but lack a specific vaccine.

*B. melitensis* Rev1 is an attenuated smooth vaccine, used in vaccination campaigns against ovine brucellosis, that protects not only against homologous *B. melitensis* infections but also against heterologous infection by *B. ovis* (8, 9). However, since it induces antibodies against O-chains, which are targets for the serological diagnosis of *B. melitensis* infection, this vaccine is banned in regions where zoonotic *B. melitensis* is considered eradicated. Accordingly, ovine brucellosis caused by rough *B. ovis* is increasing in these regions (10), which highlights the need to develop a specific vaccine against *B. ovis* infection that does not interfere with the diagnosis of brucellosis caused by smooth *Brucella* species.

Both the development of specific attenuated vaccines and the understanding of the mechanisms underlying the differences of pathogenicity and host preference observed among the species of the genus *Brucella* require a better knowledge of the bacterial components needed for infection in each species. Most works analyzing *Brucella* virulence have been performed with smooth zoonotic species (mainly with *B. melitensis* and *Brucella abortus*), although studies with *B. ovis* are increasing in number in the recent years. These studies have evidenced not only similarities but also relevant differences between *B. ovis* and smooth brucellae. Thus, some virulence factors described in smooth brucellae, such as the type IV secretion system encoded by the *virB* operon, the quorum-sensing transcriptional regulator VjbR, ß-1,2 glucans, and core LPS glycosyltransferases or pyruvate phosphate dikinase, are also required for virulence in *B. ovis* (11–13). However, some other proteins that are essential in smooth *B. melitensis* or *B. abortus* for full virulence, such as Omp10, Omp19, BepC, SP41, BacA, or the flagellar apparatus, are not required in *B. ovis* (11, 14, 15), while an ATP-binding cassette transporter, absent in the main zoonotic smooth brucellae, is a virulence factor in *B. ovis* (16).

The surface of bacterial pathogens is a key structure connecting the cell with the surrounding environment, including host cells and defense mechanisms. The LPS O-chains are essential for virulence in smooth brucellae (5, 6), and it is known that they mask other surface components (5, 17). Considering the rough nature of *B. ovis*, molecules exposed on the bacterial surface and/or regulatory mechanisms affecting its structure could be relevant actors in host–pathogen interactions, targets for the development of attenuated vaccines and related to the differences in pathogenicity and host preference that exist in the genus *Brucella*. In fact, in addition to the presence or absence of O-chains in the LPS, the *Brucella* species differ in outer membrane (OM) composition (5, 6, 18) and OM-related properties (5, 6, 19).

Among the virulence factors identified in smooth brucellae, MucR is a transcriptional regulator affecting the surface properties and virulence in *B. melitensis* and *B. abortus* (20–22), but that has not been studied in rough *B. ovis*. In this work, we have constructed a Δ*mucR* mutant in virulent *B. ovis* PA and evaluated its growth characteristics, properties related to the bacterial surface, expression of relevant genes, and virulence in cellular and animal models. Additionally, considering the high levels of HdeA—a protein that has been related to acid stress resistance in *B. abortus* (23)—detected in the Δ*mucR* mutant (see below), we have also constructed and characterized the Δ*hdeA* mutant in the genetic background of parental *B. ovis* PA and in the isogenic Δ*mucR* mutant. The results are discussed in comparison with those obtained with Δ*mucR* mutants of other brucellae.

## Materials and Methods

### Cloning Vectors, Bacterial Strains, and Culture Conditions

Plasmid pGEM-T Easy (Promega, Madison, WI, USA) was used to clone PCR products, and pCVD-KanD (11)—which does not replicate in *Brucella* spp. and confers sucrose sensitivity and kanamycin resistance—was used to construct, as described below, the recombinant plasmids containing the inactivated genes used for mutagenesis. Plasmid pBBR1MCS-2, which replicates in *Brucella* spp. and confers kanamycin resistance (24), was used for genetic complementation of the Δ*mucR* mutant with wild-type *mucR*.

*B. ovis* PA was used as a parental strain for the construction of the mutant strains described in Table 1. *B. ovis* PA-derived strains were cultured on tryptic soy agar (TSA) or broth (TSB) (Pronadisa-Laboratorios Conda, Torrejón de Ardoz, Spain) supplemented with 0.3% yeast extract (YE, (Pronadisa-Laboratorios Conda, Torrejón de Ardoz, Spain) and 5% horse serum (HS) (Gibco-Life Technologies, Grand Island, NY, USA) (TSA-YE-HS or TSB-YE-HS). When required for the construction and maintenance of the genetically engineered strains, kanamycin (50 μg/ml) or sucrose (5%) was added to the medium. *B. ovis* mutants defective in proteins of the Omp25/Omp31 family (Table 1) were previously obtained (25, 26) and used as controls in immunoblot assays. *B. ovis* strains were cultured at 37°C under a 5% CO_2_ atmosphere.

**Table 1:** Main *B. ovis* bacterial strains used in this work (intermediate bacterial strains obtained during procedures of mutagenesis are not cited).

**Strain name**	**Relevant characteristics**	**Source**
Parental strain and mutants constructed in this work		
*B. ovis* PA	Parental strain	BCCN
*B. ovis* Δ*mucR*	*mucR* deletion mutant of *B. ovis* PA	This work
*B. ovis* Δ*mucR* M2	Additional independent *mucR* deletion mutant of *B. ovis* PA	This work
*B. ovis* Δ*hdeA*	*hdeA* deletion mutant of *B. ovis* PA	This work
*B. ovis* Δ*mucR*Δ*hdeA*	Double *mucR-hdeA* deletion mutant of *B. ovis* PA	This work
*B. ovis* Δ*mucR* comp	*B. ovis* Δ*mucR* complemented *in trans* with wild-type *mucR*	This work
*B. ovis* PA mutants previously described		
*B. ovis-*pPS31OVL02M	*omp31* deletion mutant (Δ*omp31)*	(25)
*B. ovis-*pNV25OVL02M	*omp25* deletion mutant (Δ*omp25)*	(25)
B. *ovis* PNV25cA	*omp25c* deletion mutant (Δ*omp25c)*	(26)
B. *ovis* PNV25dA-com	Complemented *omp25d* deletion mutant (Δ*omp25d* comp*)*	(26)

*BCCN, Brucella Culture Collection Nouzilly (Institut National de la Recherche Agronomique, Nouzilly, France)*.

*Escherichia coli* JM109 was used for the replication of pGEM-T Easy, pBBR1MCS-2, and their derived recombinant plasmids. *E. coli* CC118 was used for the replication of pCVD-KanD and its derived recombinant plasmids. *E. coli* strains were cultured at 37°C in Luria–Bertani medium that was supplemented with 50 μg/ml ampicillin or kanamycin when required. The microbiological procedures were reviewed and approved by the Biosecurity Committee of the University of Salamanca, Spain.

### Primers, Analysis of DNA Sequences, and Construction and Genetic Complementation of *B. ovis* Mutant Strains

The primers (IDT, Leuven, Belgium) used in this work for the construction and characterization of mutant strains are listed in Table 2 and were designed according to the published genome sequence of *B. ovis* 63/290 (ATCC 25840), with GenBank accession numbers NC_009505 and NC_009504 for chromosomes I and II, respectively.

**Table 2:** Primers used in this work.

**Primer name**	**Nucleotide sequence 5^′^-3^′^^a^**	**Target gene or plasmid^**b**^**
Construction of *B. ovis* PA mutants		
mucRMUT-F	TGATGCCGTGCCGTGTGT	*mucR (BOV_0570)*
mucROVL-R	TTCGTCGTTCGTTTCCAG	*mucR (BOV_0570)*
mucROVL-F	ctggaaacgaacgacgaaGACGCCTGATTCTTCAGC	*mucR (BOV_0570)*
mucRMUT-R	TGACATCCAACAGTTCCA	*mucR (BOV_0570)*
mucR-com1	AAGGGGTGGGTTGCCATT	*mucR (BOV_0570)*
mucR-com2	TCCGATGCATCAAAGCGA	*mucR (BOV_0570)*
hdeAMUT-F	TGCAACTGCAAGCCTTGT	*hdeA (BOV_A0312)*
hdeAOVL-R	CATTTCCTTCTCCTTCGC	*hdeA (BOV_A0312)*
hdeAOVL-F	gcgaaggagaaggaaatgGTTTTCTGATCCTTCGCC	*hdeA (BOV_A0312)*
hdeAMUT-R	ACGAGCGCCCAGAAGGTA	*hdeA (BOV_A0312)*
Primers for RT-qPCR or verification of recombinant plasmids and mutants		
Universal-F	GTTTTCCCAGTCACGAC	pGEM-T Easy
Universal-R	CAGGAAACAGCTATGAC	pGEM-T Easy
mucR-R4	CCGTCTTCAAGGCAGACA	*mucR (BOV_0570)*
mucRRT-F	CATTCGTGCAGGCGAACT	*mucR (BOV_0570)*
hdeART-F	GCCAAGACCCATAAGACT	*hdeA (BOV_A0312)*
hdeART-R	GGGTTACGGTTTCGATAC	*hdeA (BOV_A0312)*
25dRT-F3	GAAAACCGCACCAATGGC	*omp25d (BOV_0115)*
NV25d-2	GATTGCCGCTGGTGATGA	*omp25d (BOV_0115)*
BOV_A0299RT-F	AGGCACTCAACTGGAAGA	*BOV_A0299*
BOV_A0299RT-R	GCCGCTATCCTTCAGTTT	*BOV_A0299*
31RT-F3	AGTCTCGAAGGCAACGCT	*omp31 (BOV_A0366)*
31RT-R3	ACGTGTGCAGGGCAGGTG	*omp31 (BOV_A0366)*
omp25 MAT	GCCGACGCCATCCAGGAA	*omp25 (BOV_0692)*
25RT-R Real	CCAACGGTGCTGGTCTT	*omp25 (BOV_0692)*
25cRT-F Real	TGAGAAACAGGGGCGAAT	*omp25c (BOV_0116)*
25cRT-R2	TCGTCGAAGCCGTAATCC	*omp25c (BOV_0116)*
blxR-F Real	TTCCCTGAATGGCTCCTT	*blxR (BOV_0183)*
blxR-R Real	CAGATTTGCGGTGCACTT	*blxR (BOV_0183)*
BOV_1296RT-F	TCAAGGCTGCCGATACTA	*BOV_1296*
BOV_1296RT-R	TCTTGCGCTGATTCTGGA	*BOV_1296*
BOV_1963RT-F2	ACTTTCAACGCCGATACG	*BOV_1963*
BOV_1963RT-R2	CAGCTTTCCGACGCTTTT	*BOV_1963*
BOV_1925RT-F	GCCTGCTCATCGAAATCA	*aqpZ (BOV_1925)*
BOV_1925RT-R	CGGGTTTACCGACGTATT	*aqpZ (BOV_1925)*
BOV_1935RT-F	TGCAGCGAGCATACAAGA	*BOV_1935*
BOV_1935RT-R	TGCCGACAGTATTACAGG	*BOV_1935*
BOV_0982RT-F	AGGAGCCGACAAAGAACA	*BOV_0982*
BOV_0982RT-R	AGCGAATTGCTCGATGGT	*BOV_0982*
fliFRT-F	TTGATGGGTGCGATCCTC	*fliF (BOV_A1051)*
fliFRT-R	CCTTGCCGATTGGAACGA	*fliF (BOV_A1051)*
fliCRT-F	CAAACTCGTCGGCTCTGA	*fliC (BOV_A1052)*
Flg1OVL-R	ATTGGCCTTGTTGTCGGA	*fliC (BOV_A1052)*
vjbR-F4	GCGCTTCTAACCCGCATC	*vjbR (BOV_A0110)*
vjbR-R Real	AACCGGGTCAATGGCAAA	*vjbR (BOV_A0110)*
virB2-F Real	TCGCGGATTCTACCTCA	*virB2 (BOV_A0062)*
virB2-R Real	TGTAACCGGACCAGATGA	*virB2 (BOV_A0062)*
FeAFtr-F	TCGACAATGGCAAGGTTC	*ftrA (BOV_A0329)*
FeAFtr-R	ATGCGCATGTTGATTGGC	*ftrA (BOV_A0329)*
bacteriofRT-F	GCCGACAAGCTGATTGA	*bfr (BOV_A0530)*
bacteriofRT-R	CGAGCTTGTCGCAGATTT	*bfr (BOV_A0530)*
BOV_1897RT-F	TTACGTCCATGCCACCAA	*BOV_1897*
BOV_1897RT-R	TCCTGCAAGATGAAACGC	*BOV_1897*
16S-RT Fw	TCTCACGACACGAGCTGACG	*16S (BOV_1586)*
16S-RT Rv	CGCAGAACCTTACCAGCCCT	*16S (BOV_1586)*

a *The primers were purchased from IDT, Leuven, Belgium. Lowercase sequences in mucROVL-F and hdeAOVL-F correspond to regions overlapping with mucROVL-R and hdeAOVL-R, respectively*.

b *The target gene is the B. ovis gene to be deleted or PCR-amplified. The primers were designed according to the published genome sequence of B. ovis 63/290 (ATCC 25840) (accession numbers NC_009505 and NC_009504 for chromosome I and II, respectively). The primers targeting 16S were those previously described (27). Primers Universal-F and Universal-R were used for sequencing the DNA insert of the pGEM-T Easy recombinant plasmids*.

GenBank accession numbers were also used to retrieve the respective DNA sequences from *B. melitensis* 16M (AE008917 and AE008918), *B. abortus* 2308 (AM040264 and AM040265), and *Brucella canis* RM6/66 (NZ_CP007758 and NZ_CP007759). Orthologs of the analyzed genes were identified at the Kyoto Encyclopedia of Genes and Genomes (https://www.kegg.jp), and multiple nucleotide sequence alignments were performed with Clustal Omega at the European Bioinformatics Institute (https://www.ebi.ac.uk/Tools/msa/clustalo/).

For non-polar deletion of *mucR* in *B. ovis* PA, the wild-type chromosomal gene was replaced by the inactivated gene, following a procedure previously described (11, 15). Briefly, two PCR reactions were performed to amplify the 5′ and 3′ ends of *mucR*, together with about 700 bp located upstream or downstream the gene, respectively. Amplification of the 5′ end was performed with *B. ovis* PA DNA and primers mucRMUT-F and mucROVL-R, while the 3′ end was amplified with primers mucROVL-F and mucRMUT-R (Table 2). The two PCR products were fused by an overlapping PCR with primers mucRMUT-F and mucRMUT-R through the overlapping section of primers mucROVL-F and mucROVL-R (Table 2). The amplicon was cloned in plasmid pGEM-T Easy and transformed in *E. coli* JM109. The correct nucleotide sequence of the insert cloned in the resulting pNVmucR01 recombinant plasmid—that contains the *mucR* gene of *B. ovis* PA almost completely deleted and adjacent DNA to both sides of the gene—was verified by automated Sanger sequencing with primers Universal-F and Universal-R (Table 2) at the DNA sequencing facility of the Universidad de Salamanca, Spain. The insert of pNVmucR01 was extracted by digestion with *SphI* and *SacI* restriction enzymes and cloned into plasmid pCVDKan-D digested with the same enzymes. The resulting plasmid pNVmucR02, containing the defective *mucR* gene together with the *sacB* gene that confers sensitivity to sucrose and a kanamycin resistance cassette, was replicated in *E. coli* CC118 and subsequently introduced in parental *B. ovis* PA by electroporation. The bacteria were cultured in TSA-YE-HS plates supplemented with Kan to select an intermediate strain with the entire plasmid integrated in the chromosome after a single homologous recombination event occurred through one of the *mucR* ends. The intermediate strain, which is resistant to Kan and contains a copy of wild-type *mucR* and one copy of Δ*mucR*, was cultured in the presence of sucrose to select for the second homologous recombination event that leads either to a revertant strain recovering the parental genotype or to the desired Δ*mucR* mutant and the loss of the plasmid. Differentiation between both *B. ovis* PA strains was performed by PCR with specific primers annealing inside and/or outside the deleted fragment (pairs mucRMUT-F + mucRMUT-R and mucRMUT-F + mucR-R4) (Table 2). Two intermediate strains selected from two independent electroporation events were selected to obtain two independent mutant strains for confirmative studies. The Δ*mucR* mutant was complemented *in trans* with plasmid pNVmucRcom01, which is pBBR1MCS-2 bearing wild type *mucR* of *B. ovis* PA amplified with primers mucR-com1 and mucR-com2 (Table 2).

The Δ*hdeA* mutant of *B. ovis* PA was obtained with the same procedure but using the specific primers listed in Table 2. The pNVhdeA02 plasmid was electroporated in parental *B. ovis* PA to obtain the Δ*hdeA* mutant and in the Δ*mucR* mutant to obtain the Δ*mucR*Δ*hdeA* double mutant.

### Growth, Autoagglutination, and Susceptibility Assays

The growth characteristics of mutant strains were evaluated in comparison with those of the parental strain *B. ovis* PA. Bacterial suspensions of optical density values at 600 nm (OD_600_) of 0.2 were prepared in phosphate-buffered saline (PBS). Serial dilutions were plated in triplicate on TSA-YE-HS plates that were then incubated for 10 days at 37°C in a 5% CO_2_ atmosphere. The colony size was periodically checked, and the number of colony-forming units (CFU)/ml corresponding to the OD_600_ score of 0.2 was determined and used for further experiments.

Growth curves were determined in TSB-YE-HS inoculated with 2.5 × 10^8^ CFU/ml of each *B. ovis* strain and incubated under agitation at 120 rpm at 37°C and 5% CO_2_. The OD_600_ scores were periodically determined, and the number of CFU/ml was evaluated at several time points by platting serial dilutions on TSA-YE-HS plates. To evaluate susceptibility to hypersaline medium, TSA-YE-HS containing 1 M NaCl was inoculated with 2.5 × 10^8^ CFU/ml of each bacterial strain and incubated under agitation at 120 rpm at 37°C and 5% CO_2_ for 24 h when the number of CFU/ml was determined. Susceptibility to acid pH was evaluated similarly in TSB-YE-HS adjusted to pH 4.4 and inoculated with 5 × 10^8^ CFU/ml of each bacterial strain. The results were presented as means ± SD of three assays.

Autoagglutination ability was evaluated as previously described (11, 26) by determining the evolution of OD_600_ values—in static incubation at room temperature—of bacterial suspensions in TSA-YE-HS of initial OD_600_ scores of 0.8. Susceptibility test to polymyxin B (10 mg/ml), sodium deoxycholate (10 mg/ml), sodium dodecyl sulfate (SDS, 10 mg/ml), Tween 20 (10%), 3-[(3-cholamidopropyl)dimethylammonio]-1-propanesulfonate (CHAPS, 100 mg/ml), 2,2′-dipyridyl (0.1 M), and hydrogen peroxide (H_2_O_2_, 7.5%) (Sigma Aldrich, St. Louis, MO, USA) was performed in a disc assay. The bacterial suspensions (100 μl containing 10^8^ CFU) were spread on TSA-YE-HS plates. Then, a paper disc (diameter of 9 mm) was deposited on the center of the plate and soaked with 20 μl of each agent. The diameter of the growth inhibition zone was measured after 7 days of incubation at 37°C and 5% CO_2_ (four diameter scores were considered to establish the mean value for each plate). The results were presented as means ± SD of three assays.

### Protein and Immunological Techniques

SDS-polyacrylamide gel electrophoresis (SDS-PAGE) was performed, as previously described (18), with whole-cell bacterial lysates and using pre-stained protein marker VI (Applichem-Panreac, Barcelona, Spain) as protein standard. The samples were resolved in a Protean II xi cell (Bio-Rad, Hercules, CA, United States) using 14% acrylamide/bisacrylamide gels. After the electrophoresis step, the protein bands were stained with Coomassie blue or transferred to nitrocellulose with a semidry electroblotter (Amersham, GE Healthcare, Little Chalfont, United Kingdom) for the analysis of reactivity with antibodies against surface antigens.

The proteomic analysis of protein bands excised from SDS-PAGE gels was performed by MALDI-TOF or LC–MS/MS, after trypsin in-gel digestion, in the proteomics facility of Centro de Investigación del Cáncer, Salamanca, Spain, following its standardized procedures.

Immunoblot was performed, as described before (18, 28), with rabbit antibodies raised previously against Omp31b, Omp25c, or Omp25d recombinant outer membrane proteins of *Brucella* spp. (18), a goat anti-rabbit IgG-peroxidase conjugate as secondary antibody, and 4-chloro-1-naphthol (Sigma Aldrich, St. Louis, MO, USA) as substrate for peroxidase. Before immunoblotting, the proteins transferred to the nitrocellulose membrane were stained with Ponceau S (Sigma Aldrich, St. Louis, MO, USA) to check the protein load (that was similar in all tested strains).

### Quantitative Reverse-Transcription PCR

For relative quantification of transcripts by real-time reverse transcription-PCR (RT-qPCR), bacterial strains were incubated in TSB-YE-HS for 15 h (exponential growth phase), and RNA was extracted using the E.Z.N.A.® Bacterial RNA Kit (Omega Bio-tek Inc., Norcross, GA, USA). Contaminant DNA was removed by DNaseI, RNase-free treatment (Thermo Fisher Scientific, Vilnius, Lithuania), and cDNA synthetized from 1 μg of RNA with the NZY first-strand cDNA synthesis kit (NZYTech Lda., Lisboa, Portugal). A reaction without retrotranscriptase was also settled to be used in RT-PCR reactions as control of DNA absence. Real-time reactions were performed, in a StepOnePlus^TM^ apparatus (Applied Biosystems, Foster City, USA), on cDNA obtained from *B. ovis* PA, the Δ*mucR* mutant, and the Δ*mucR* mutant complemented with wild-type *mucR*. The primer pairs listed in Table 2 and NZYSupreme qPCR Green Master Mix (2x) ROX plus (NZYTech Lda., Lisboa, Portugal) were used in the amplification reactions. Two independent biological samples with three technical replicates for each strain and primer pair were analyzed. Calculation of relative expression was performed with the StepOne^TM^ software v2.3 (2^−Δ*ΔCt*^ method). *B. ovis* PA and *16S* were used as reference strain and gene, respectively, and the results were expressed as mean ± SD of the log_2_ of the relative quantity (log_2_RQ).

### Virulence Assays

J774A.1 murine macrophages were used to evaluate the intracellular behavior of *B. ovis* strains in phagocytic cells as previously described (14). Briefly, 2 × 10^4^ macrophages per well were incubated for 24 h in 96-well plates at 37°C and 5% CO_2_. The macrophages were then infected with each *B. ovis* strain at a multiplicity of infection of 1:200. After an incubation period of 2 h, extracellular bacteria were killed with gentamycin, macrophages lysed, and the number of intracellular bacteria determined in three wells per strain by plating serial dilutions on TSA-YE-HS (t0). Intracellular bacteria were also determined at 20 and 44 h later (t20 and t44) in three wells per strain, where infected macrophages had been maintained in the presence of gentamycin. The results were expressed as means ± SD (*n* = 3) of the log_10_ CFU/well values at each time point.

Virulence in mice was evaluated in 6-week-old female BALB/c mice (Charles River Laboratories, Chatillon-sur-Chalaronne, France). Mice received a week before were inoculated intraperitoneally with 10^6^ or 10^8^ CFU of each bacterial strain in 0.2 ml PBS. At several post-infection (p.i.) time points, CFU were determined in spleen, in five mice per group, as described before (29). The results were expressed as means ± SD (*n* = 5) of the log_10_ CFU/spleen values at each time point. The mice experiments were designed according to the Spanish and European legislation for research with animals (RD 53/2013 and directive 2010/63/EU).

### Statistical Analysis

Statistical analysis was performed by one-way ANOVA and Fisher's LSD using the GraphPad Prism 7 Software (GraphPad Software Inc., San Diego, CA, United States). A 99% confidence interval was considered for statistically significant differences (*P* < 0.01).

## Results

### HdeA, Omp25d, and BOV_A0299 Are Over-translated in *B. ovis* PA Δ*mucR*

**Figure 1:**
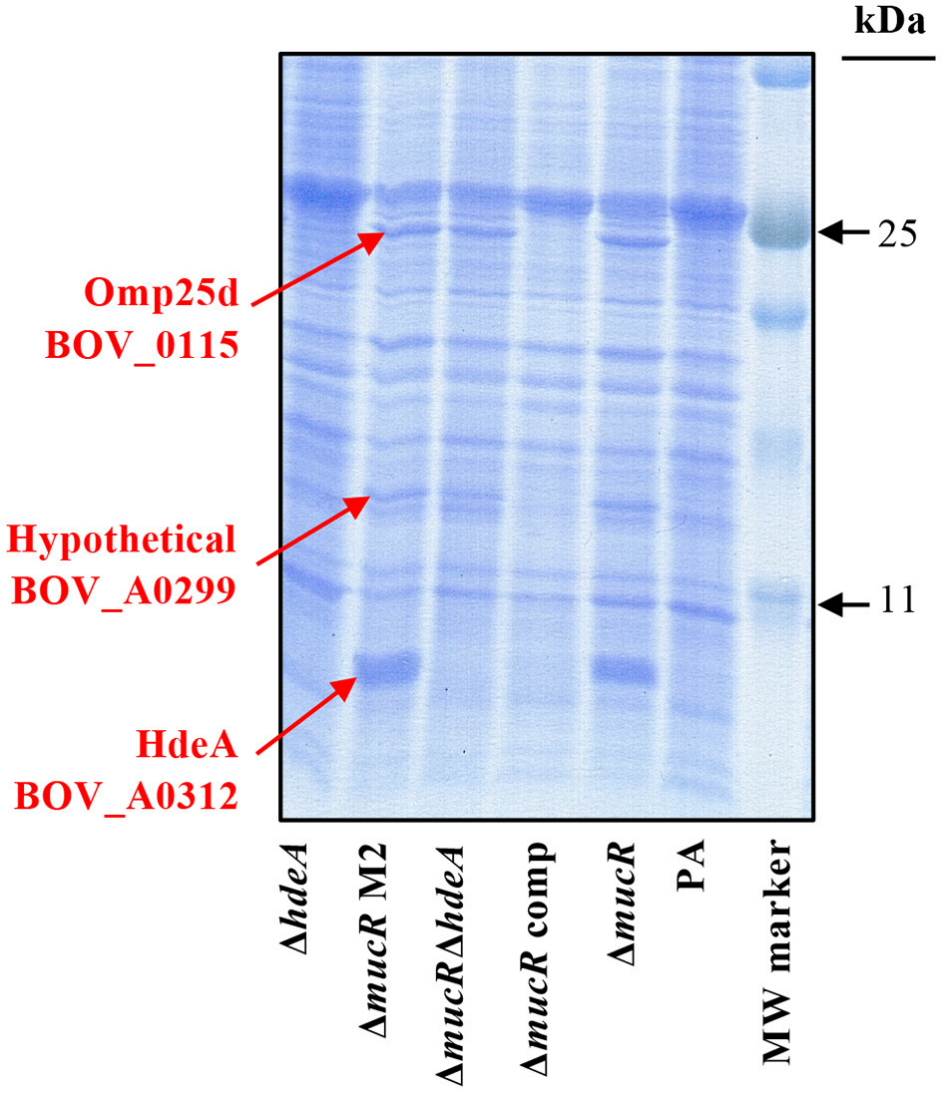
Protein profile in SDS-PAGE of the *B. ovis* PA mutants. Proteins were stained with Coomassie blue after electrophoresis. Overrepresented proteins in the Δ*mucR* and Δ*mucR*Δ*hdeA* mutants, which were excised from the gel for identification, are highlighted in red.

A first proteomic analysis by SDS-PAGE was performed with parental *B. ovis* PA, the Δ*mucR* mutant and the Δ*mucR* mutant complemented *in trans* with wild-type *mucR*. When compared to the parental strain, three proteins of the Δ*mucR* mutant showed increased levels that recovered those of the parental strain after complementation with wild-type *mucR* (Figure 1). The results were also reproduced in a second Δ*mucR* mutant (Δ*mucR* M2) (Figure 1), which was obtained from another independent mutagenesis procedure and was included in some analysis to obtain additional confirmation of results. The three protein bands were excised from the gel and identified by the proteomics facility of Centro de Investigación del Cáncer, Salamanca, Spain. A protein of low molecular mass showed a remarkable intensity in the Δ*mucR* mutant (Figure 1) and was identified as the product of the gene BOV_A0312, which codes for a protein annotated as acid-activated periplasmic chaperone HdeA in the genome sequence of *B. ovis* 63/290 (GenBank accession number NC_009504) and that contributes to acid resistance in *B. abortus* 2308 (23). Another protein band matched Omp25d (BOV_0115) (Figure 1), a member of the *Brucella* Omp25/Omp31 family of OMPs that has never been detected in parental *B. ovis* PA and was only detected in a Δ*omp25d* mutant complemented *in trans* that overexpresses *omp25d* (18). The third overproduced protein, located between Omp25d and HdeA in the SDS-PAGE gel, was identified as the product of gene BOV_A0299, which corresponds to a hypothetical protein lacking homology with proteins of known function.

Considering that brucellae are intracellular bacteria facing acidic conditions in the phagocyte (30) and that HdeA, described as chaperone activated under acidic conditions, is highly represented in the Δ*mucR* mutant (Figure 1) and has never been studied in *B. ovis*, the *hdeA* deletion mutant was constructed in *B. ovis* PA and in the Δ*mucR* mutant. Both single Δ*hdeA* and double Δ*mucR*Δ*hdeA* mutants were included in further experiments.

As expected, overproduction of HdeA was not detected when *hdeA* was deleted from the Δ*mucR* mutant, but the resulting double Δ*mucR*Δ*hdeA* mutant maintained the increased levels of Omp25d and BOV_0299 (Figure 1). No apparent differences in the SDS-PAGE protein profile were observed between the parental strain and the single Δ*hdeA* mutant, although it must be considered that a discrete HdeA band is not detected in the parental strain (Figure 1).

### *B. ovis* PA Δ*mucR* and Δ*mucR*Δ*hdeA*, but Not Δ*hdeA*, Have Defective *in vitro* Growth

**Figure 2:**
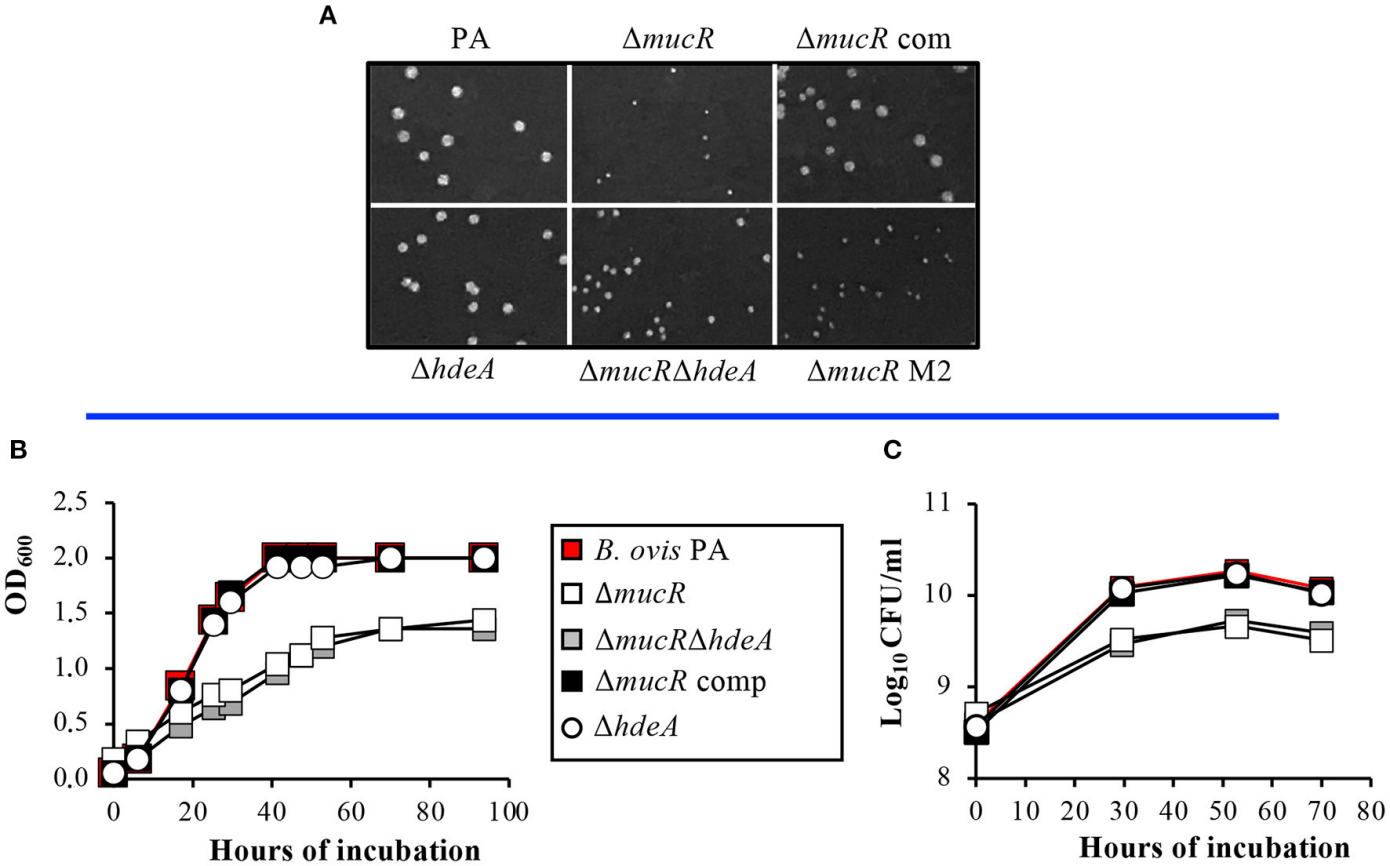
Growth characteristics of the *B. ovis* PA mutants. Colony size in TSA-YE-HS plates was monitored daily and recorded after 6 days of incubation **(A)**. Growth in TSB-YE-HS liquid medium was evaluated by measuring the evolution over time in OD_600_ **(B)** and CFU/ml **(C)** values.

The colony size of mutant strains and parental *B. ovis* PA in TSA-YE-HS solid medium was monitored daily and recorded after 6 days (Figure 2A). Colonies of the Δ*mucR* mutant were significantly smaller than those of *B. ovis* PA, and complementation of the mutant with wild-type *mucR* restored the parental phenotype. The Δ*mucR*Δ*hdeA* double mutant had slightly bigger colonies than the Δ*mucR* mutant, but not as big as those of the parental strain (Figure 2A). On the contrary, the single deletion of *hdeA* in *B. ovis* PA did not have an apparent effect on colony size (Figure 2A).

The growth deficiencies of the *mucR* mutants were also evident in TSB-YE-HS liquid medium since the Δ*mucR* and Δ*mucR*Δ*hdeA* mutants had reduced replication rates, and their OD_600_ and log_10_ CFU values in the stationary phase were significantly lower than those obtained with the parental strain and the Δ*hdeA* mutant (Figures 2B,C).

### *B. ovis* PA Δ*mucR* and Δ*mucR*Δ*hdeA*, but Not Δ*hdeA*, Have Altered Outer Membrane-Related Properties

A disc assay was used to determine the susceptibility of the mutant strains derived from *B. ovis* PA to several compounds related to outer membrane properties and/or survival in the host. No statistically significant differences were found between parental *B. ovis* PA and the mutant strains regarding susceptibility to H_2_O_2_ or the high-affinity iron chelator 2,2′-dipyridyl (Table 3). On the contrary, the Δ*mucR* single mutant and the Δ*mucR*Δ*hdeA* double mutant were more susceptible (*P* < 0.001) to the cationic peptide polymyxin B and the detergents sodium deoxycholate, sodium dodecyl sulfate, Tween 20, and CHAPS (Table 3). Complementation *in trans* of the Δ*mucR* mutant with wild-type *mucR* restored the parental phenotype (Table 3).

**Table 3:** Susceptibility pattern of the *B. ovis* PA mutants in a disc assay (discs—diameter of 9 mm—were deposited on the center of TSA-YE-HS plates containing 10^8^ CFU of each bacterial strain).

	**Mean** **±SD of the inhibition zone diameter (cm) after exposure**						
**Strain**	**Polymyxin B (10 mg/ml)**	**DOC** **(10 mg/ml)**	**SDS (10 mg/ml)**	**Tween-20** **(10%)**	**CHAPS (100 mg/ml)**	**H_**2**_O_**2**_** **7.5%**	**2,2^′^-dipyridyl (0.1 M)**
*B. ovis* PA	2.59 ± 0.03	2.13 ± 0.07	2.23 ± 0,02	2.09 ± 0.02	2.56 ± 0.12	4.05 ± 0.15	3.46 ± 0.19
Δ*mucR*	3.32 ± 0.14^*^	3.33 ± 0.07^*^	2.57 ± 0.08^*^	2.70 ± 0.14^*^	3.89 ± 0.12^*^	4.15 ± 0.43	3.46 ± 0.33
Δ*mucR* comp	2.53 ± 0.08	1.94 ± 0.09	2.35 ± 0.07	2.31 ± 0.05	2.50 ± 0.07	4.04 ± 0.13	3.41 ± 0.41
Δ*mucR*Δ*hdeA*	3.04 ± 0.09^*^	3.10 ± 0.06^*^	2.58 ± 0.05^*^	2.66 ± 0.02^*^	3.84 ± 0.12^*^	4.27 ± 0.33	3.75 ± 0.08
Δ*hdeA*	2.58 ± 0.04	2.15 ± 0.14	2.15 ± 0.05	2.00 ± 0.07	2.86 ± 0.02	4.09 ± 0.12	3.54 ± 0.05

*Discs were soaked with 20 μl of each compound, and the diameter of the growth inhibition area was measured after a period of incubation of 7 days*.

*The results are mean ± SD of three assays. Statistically significant differences (P < 0.01), when compared to the parental strain, are marked with an asterisk*.

**Figure 3:**
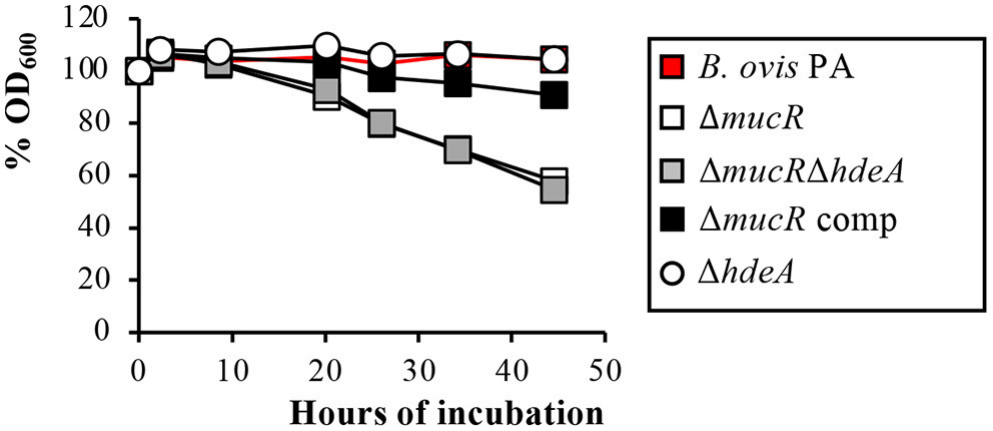
Autoagglutination assay with the *B. ovis* PA mutants. The percentages of the OD_600_ values with respect to the initial value of 0.8 were recorded over time and are presented in the figure.

Additionally, the Δ*mucR* single and double mutants showed autoagglutination ability indicative of surface alterations (Figure 3), but no significant differences were found among strains regarding survival to exposure for 24 h to hypersaline or acid pH conditions (Supplementary Table S1).

### Immunodetection of Omp25d and Major OMPs of the Omp25/Omp31 Family

**Figure 4:**
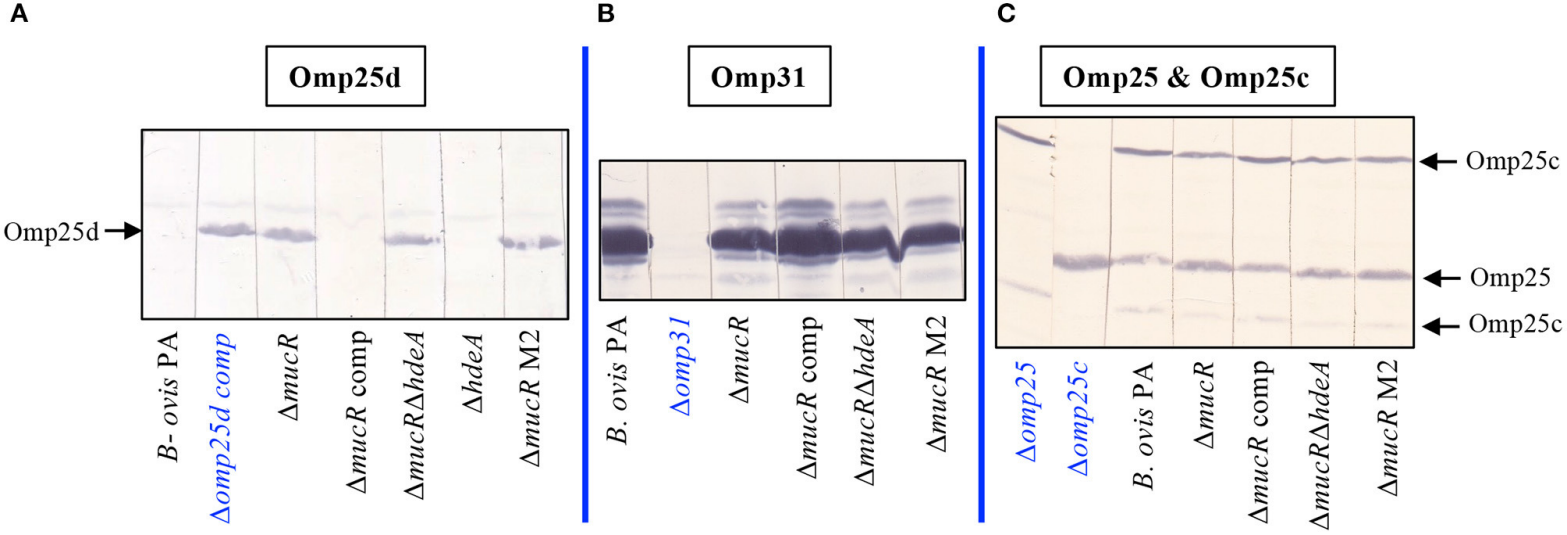
Detection of Omp25d and major outer membrane proteins of the Omp25/Omp31 family in the *B. ovis* PA mutants. Omp25d **(A)**, Omp31 **(B)**, Omp25, and Omp25c **(C)** were detected by immunoblot after SDS-PAGE resolution of whole-cell lysates. Previously characterized rabbit sera raised against recombinant proteins of the Omp25/Omp31 family (18) were used as primary antibodies. The anti-Omp25c serum allows the simultaneous detection of Omp25 and Omp25c because of cross-reactivity due to the amino acid sequence similarity between both proteins (18). Mutant *B. ovis* PA strains defective in Omp31, Omp25, or Omp25c obtained before (25, 26) were used as negative control strains for each protein. Since, as previously described, Omp25d is not detected in parental *B. ovis* PA (18), an *omp25d* mutant complemented in trans with wild-type *omp25d* and that overproduces Omp25d (18, 26) was used as the positive control strain for this protein. The *omp25d* mutant was not included since *B. ovis* PA behaves as the negative control for Omp25d.

Confirmation of Omp25d overproduction in the Δ*mucR* and Δ*mucR*Δ*hdeA* mutants was obtained by immunoblot with rabbit sera raised previously against recombinant Omp25d (18). Both mutant strains developed a protein band of identical molecular mass to that detected in a *B. ovis* PA Δ*omp25d* mutant complemented *in trans* with *omp25d* and that overproduces Omp25d (18) (Figure 4A). As expected according to previous reports, Omp25d was not detected by immunoblot in parental *B. ovis* PA (18), and this phenotype was recovered in the Δ*mucR* mutant complemented with *mucR* (Figure 4A). Omp25d was also undetectable in the Δ*hdeA* mutant (Figure 4A), which is in accordance with the SDS-PAGE protein profile (Figure 1).

Considering the overproduction of Omp25d detected in the Δ*mucR* and Δ*mucR*Δ*hdeA* mutants and that members of the *Brucella* spp. Omp25/Omp31 seem to be tightly balanced (18), these mutants were evaluated in immunoblot (Figures 4B,C) with rabbit sera that allow the immunological detection of Omp31, Omp25, and Omp25c major OMPs (18). Except for the Δ*omp31* mutant of *B. ovis* PA used as control in this assay (25), the characteristic multiple band pattern of Omp31 was detected in all mutant strains tested, although Omp31 appeared to be more abundant in the parental strain and the complemented Δ*mucR* mutant (Figure 4B). Although the multiple band profile of Omp31 makes it difficult to estimate its quantity by SDS-PAGE, a lower amount of Omp31 in the Δ*mucR* and Δ*mucR*Δ*hdeA* mutants would be in accordance with the transcriptomic results described below. Omp25c and Omp25 were also detected in the mutant strains (Figure 4C) by reactivity with an anti-Omp25c sera that cross-react with Omp25, thus allowing the detection of both proteins (18). Differentiation between Omp25 and Omp25c bands was performed according to the reactivity profile of the Δ*omp25* and Δ*omp25c* control strains previously obtained. Although deletion of *mucR* in *B. ovis* PA did not lead to important alterations in the levels of Omp25 and Omp25c as visualized by immunoblot (Figure 4C), subtle differences seem to exist among strains (single and double Δ*mucR* mutants seem to have a more intense Omp25 band and less intense Omp25c bands) that would also correlate with the RT-qPCR results described below.

### Δ*mucR* Mutants of *B. ovis* PA, *B. melitensis* 16M, *B. abortus* 2,308, and *B. canis* RM6/66 Display Differential Transcriptional Patterns

According to the results obtained in SDS-PAGE, HdeA and BOV_A0299 were overproduced in the Δ*mucR* mutant of *B. ovis* PA (Figure 1), suggesting that the corresponding genes are upregulated in this mutant. However, the analysis of previous works performed with smooth *B. melitensis* 16M and *B. abortus* 2308 showed that orthologs of *BOV_A0299* were not listed among the genes regulated by MucR and that *hdeA* was not cited as over-transcribed in the Δ*mucR* mutant of *B. abortus* 2308 (Table 4). Considering these differences among species, a transcriptomic analysis of the *B. ovis* PA Δ*mucR* mutant and the complemented strain was performed in comparison with the parental strain. A panel of 19 genes was selected according to the protein profile detected in SDS-PAGE and immunoblot in the *B. ovis* PA mutants (Figures 1, 4) and to the transcriptome of Δ*mucR* mutants analyzed in smooth *Brucella* species (20, 31). Data of the Δ*mucR* mutant of *B. canis* RM6/66 described during the preparation of this manuscript (32) were also included in the interspecies comparative analysis summarized in Table 4.

**Table 4:** Genes of *B. ovis* analyzed in this work and their orthologs in *Brucella* strains with available Δ*mucR* mutants.

***Brucella*** **strains**				**Protein annotation in *B. ovis* 63/290 genome** **(gene)**
***B. ovis*** **63/290**	***B. abortus* 2308**	***B. melitensis*** **16M**	***B. canis* RM6/66**	
*BOV_A0312*	*BAB2_0862*	*BMEII0906*	*DK60_2070*	Acid-activated periplasmic chaperone HdeA (gene *hdeA*)
*BOV_0115*	*BAB1_0115*	*BMEI1830*	*DK60_225*	Porin family protein (gene *omp25d*)
*BOV_A0299*	*BAB2_0880*	*BMEII0924*	*DK60_2056*	Hypothetical protein
*BOV_0183*	*BAB1_0190*	*BMEI1758*	*DK60_291*	LuxR family transcriptional regulator (gene *blxR* or *babR*)
*BOV_1296*	*BAB1_1355*	*BMEI0668*	*DK60_1374*	Calcium-binding protein
*BOV_1963*	*BAB1_2041*	*BMEI0030*	*DK60_2016*	DUF4354 family protein
*BOV_1935*	*BAB1_2010*	*BMEI0062*	*DK60_1983*	Hypothetical protein
*BOV_1925*	*BAB1_2001*	*BMEI0070*	*DK60_1976*	Aquaporin Z (gene *aqpZ*)
*BOV_0982*	*BAB1_1035*	*BMEI0948*	*DK60_1068*	Hypothetical protein
*BOV_A1051*	*BAB2_1105*	*BMEII0152*	*DK60_2830*	Flagellar M-ring protein FliF (gene *fliF*)
*BOV_A1052*	*BAB2_1106*	*BMEII0150*	*DK60_2831*	Flagellin FliC (gene *fliC*)
*BOV_A0110*	*BAB2_0118*	*BMEII1116*	*DK60_3002*	QS-dependent transcriptional regulator VjbR (gene *vjbR*)
*BOV_A0366*	DELETED	*BMEII0844*	*DK60_2150*	Porin family protein (gene *omp31*)
*BOV_0692*	*BAB1_0722*	*BMEI1249*	*DK60_770*	Porin family protein (gene *omp25*)
*BOV_0116*	*BAB1_0116*	*BMEI1829*	*DK60_226*	Porin family protein (gene *omp25c*)
*BOV_A0062*	*BAB2_0067*	*BMEII*002^a^	*DK60_2955*	TrbC/VirB2 family protein (gene *virB2*)
*BOV_A0530*	*BAB2_0675*	*BMEII0704*	*DK60_2276*	Bacterioferritin (gene *bfr*)
*BOV_A0329*	*BAB2_0840*	*BMEII0885*	*DK60_2106*	Iron transporter (gene *ftrA*)
*BOV_1897*	*BAB1_1973*	*BMEI0095*	*DK60_1949*	7-Cyano-7-deazaguanine synthase QueC (gene *queC*)

*Upregulated and downregulated genes are presented in red and blue, respectively. The table was constructed considering the results obtained in this work with the B. ovis PA ΔmucR mutant and the results previously reported for the ΔmucR mutants of B. melitensis 16M, B. abortus 2308, and B. canis RM6/66 (20, 21, 31, 32)*.

a *virB2 is not listed as differentially expressed in the B. melitensis 16M ΔmucR mutant, but several other genes of the virB operon are downregulated in this strain (31)*.

**Figure 5:**
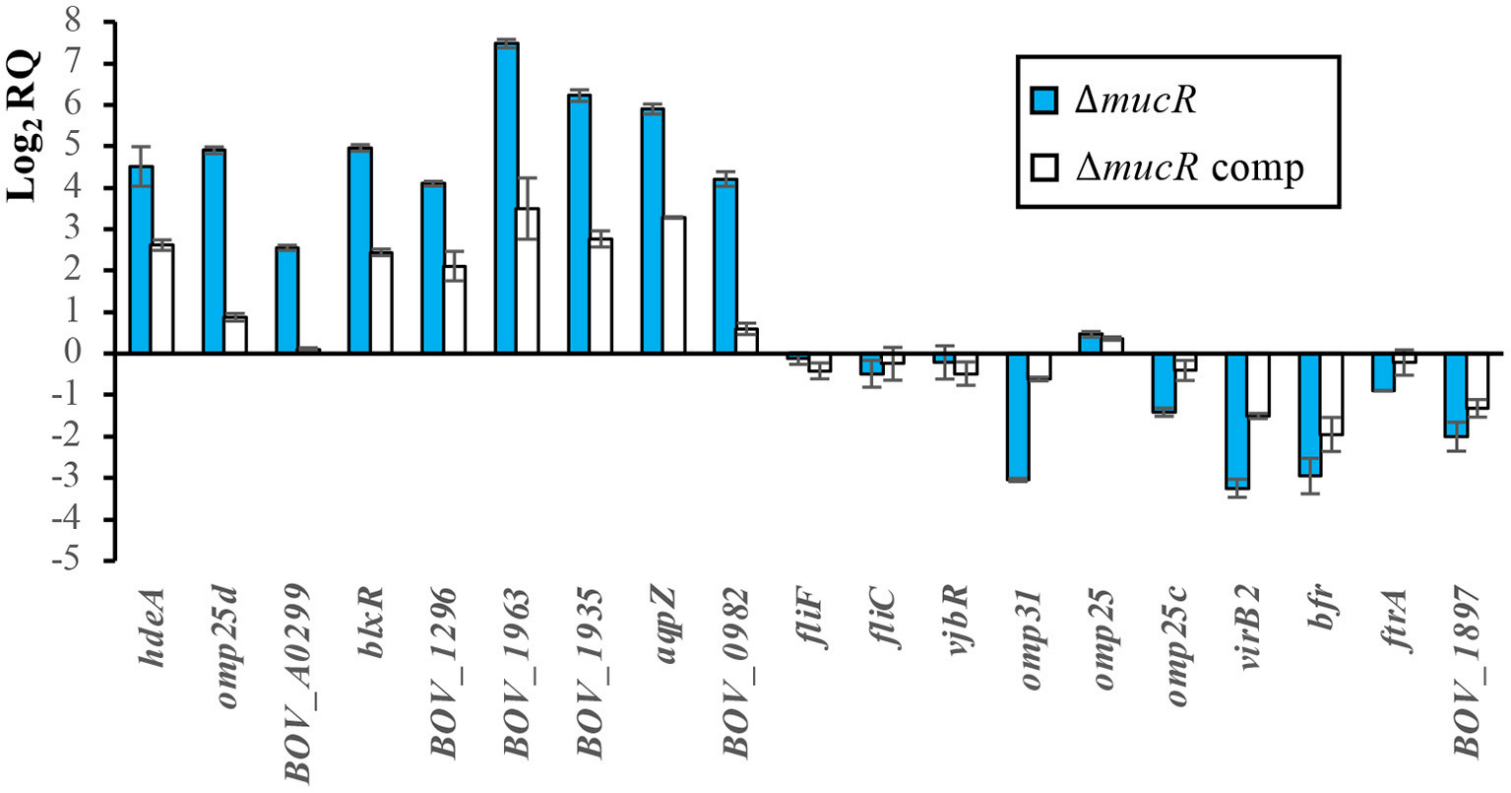
Relative quantification of transcripts of selected genes by RT-qPCR. The relative expression of each gene was evaluated by the 2^−Δ*ΔCt*^ method with the StepOne^TM^ software v2.3 and using *B. ovis* PA and *16S* gene as reference strain and gene, respectively. The results are presented as means ± SD of the log_2_ of the relative quantities (log_2_RQ) of two biological samples with three technical replicates each (genes of parental *B. ovis* PA, which was used as reference strain for relative quantification of transcripts, have a log_2_RQ value of 0).

In accordance with the proteomic results (Figure 1), transcripts for genes *hdeA, omp25d*, and *BOV_A0299* were overrepresented in the Δ*mucR* mutant of *B. ovis* PA when compared to the parental strain and the complemented mutant (Figure 5); the results were also reproduced in *B. canis* RM6/66 (Table 4). Considering the results described in *B. abortus* 2308 (20, 33), genes *BOV_0982* and *BOV_0183*, the latter coding for the LuxR family quorum sensing transcriptional regulator *blxR* (34) also named *babR* (35), were also included in the transcriptomic analysis. In *B. abortus* 2308, overexpression of the corresponding orthologs *BAB1_0190* and *BAB1_1035* in the Δ*mucR* mutant has been associated to the demonstrated direct binding of MucR to their promoter region (20, 33). Both genes were also upregulated in the Δ*mucR* mutant of *B. ovis* PA (Figure 5) and in *B. canis* RM6/66 (Table 4). However, the *B. melitensis* 16M ortholog of *BOV_0982* (*BMEI0948*) does not appear among the upregulated genes in the Δ*mucR* mutant (31) (Table 4).

Four other genes (orthologs of genes *BOV_1296, BOV_1963, BOV_1935*, and *BOV_1925*) upregulated in either *B. melitensis* or *B. abortus* Δ*mucR* mutants (but not simultaneously in both strains) were also found upregulated in the Δ*mucR* mutant of *B. ovis* (Figure 5) and *B. canis* (32) (Table 4). On the contrary, flagellar genes *fliF* and *fliC*, which are upregulated in the Δ*mucR* mutants of *B. melitensis* 16M and *B. canis*, maintain the transcription level of the parental strain in the *B. ovis* PA mutant (Figure 5), and no flagellar genes are listed among the upregulated genes in the *B. abortus* mutant (20) (Table 4). More interspecies differences in the transcriptome of *Brucella* Δ*mucR* mutants were observed regarding *vjbR*, which codes for a quorum sensing-related regulator acting as activator of flagellar and *virB* genes in *B. melitensis* (36) and that is required for virulence in *Brucella* spp. (11, 36, 37). Expression patterns dependent on *Brucella* species were also detected for genes encoding surface antigens of the Omp25/Omp31 family, for *queC*, and for virulence genes reported as downregulated in Δ*mucR* mutants of smooth *B. melitensis* 16M or *B. abortus* 2308 (20, 31), such as the *virB* operon encoding the type IV secretion system or iron homeostasis genes *bfr* and *ftrA* (Figure 5, Table 4).

### *B. ovis* PA Δ*mucR* and Δ*mucR*Δ*hdeA*, but Not Δ*hdeA*, Have Attenuated Virulence in Macrophages and Mice

**Figure 6:**
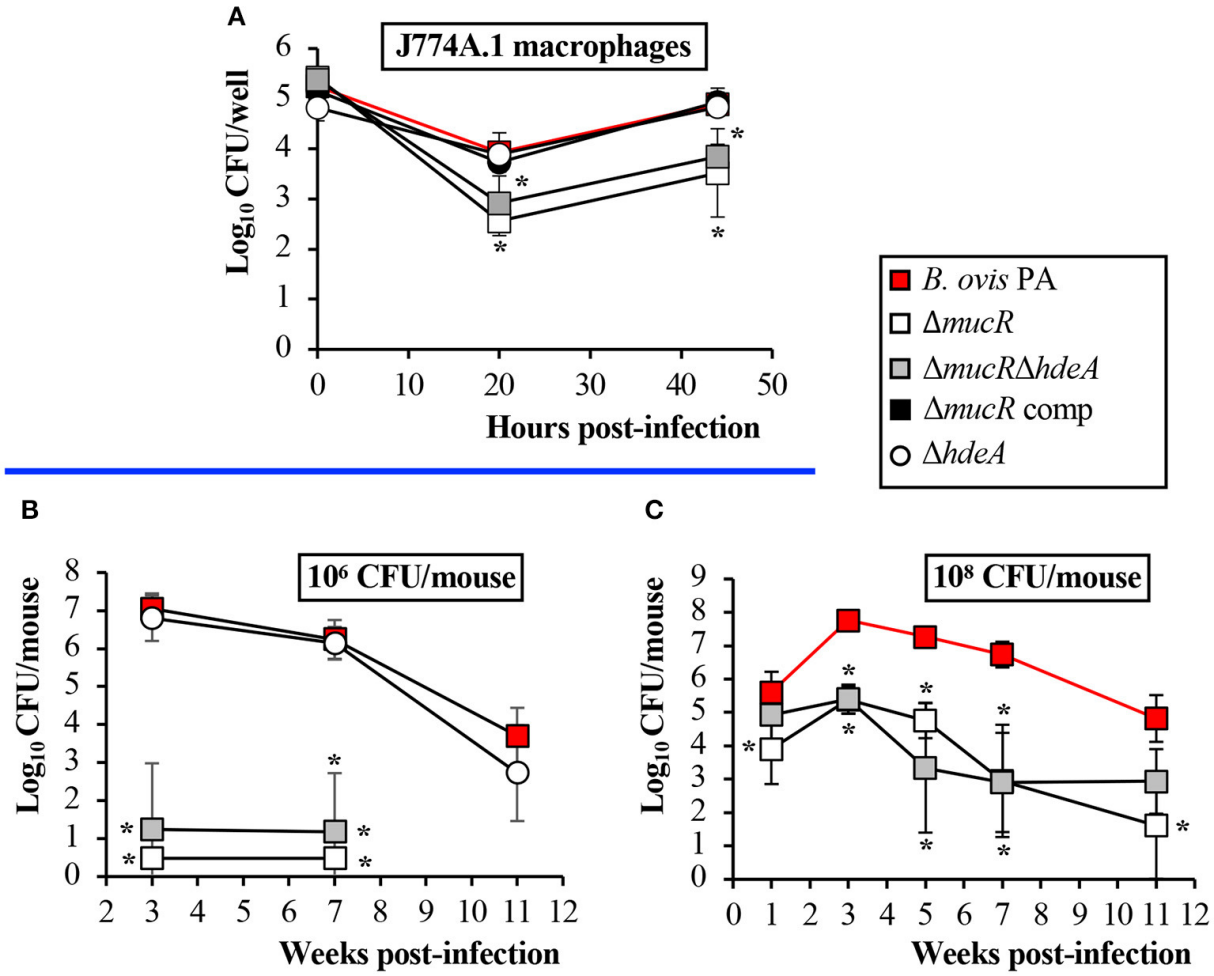
Virulence of the *B. ovis* PA mutants in J774A.1 macrophages **(A)** and mice inoculated intraperitoneally with 10^6^ CFU **(B)** or 10^8^ CFU **(C)**. Results in the macrophages are expressed as means ± SD (*n* = 3) of the log_10_ CFU/well at each time point. For evaluation of virulence in mice, bacterial numbers were determined in spleen at several time points. The results are presented as means ± SD (*n* = 5) of the log_10_ CFU/spleen. Statistically significant differences (*P* < 0.01) with the parental strain are marked with an asterisk.

Murine J774A.1 macrophages were used to determine the ability of the *B. ovis* PA mutants obtained in this work to internalize, survive, and replicate within phagocytic cells. While the Δ*hdeA* mutant behaved as the parental strain, the Δ*mucR* and Δ*mucR*Δ*hdeA* mutants internalized similarly to the parental strain but showed increased killing at t20. Both mutants were able to replicate thereafter, but intracellular counts at t44 were in the order of 1 log unit lower than those obtained with the parental strain (Figure 6A).

For a first evaluation in the mouse model, the mice were inoculated with 10^6^ CFU of the parental strain or the isogenic mutants obtained in this work. Bacterial counts in the spleen were determined at weeks 3 and 7 p.i., time points that correspond to the peak of infection in mice for *B. ovis* PA (acute phase of infection) and to the plateau of the chronic phase (25). The Δ*hdeA* mutant did not show statistically significant differences with the parental strain even at week 11 p.i., an additional point of analysis included for this mutant (Figure 6B). On the contrary, spleens of mice inoculated with the Δ*mucR* and Δ*mucR*Δ*hdeA* mutants were free of infection, except for one mouse at each sampling point of the group inoculated with *B. ovis* PA Δ*mucR*Δ*hdeA* (Figure 6B). A second experiment was performed in mice inoculated with 10^8^ CFU—a dose usually employed for protection experiments with *B. ovis* attenuated vaccines (13, 25, 29)—in which bacterial splenic colonization was monitored from week 1 to 11 p.i. In these conditions, the Δ*mucR* mutants produced a persistent infection but with bacterial splenic colonization levels that were significantly lower than those observed with the parental strain (Figure 6C).

## Discussion

In the last decade, the regulatory network of the MucR transcriptional regulator has been depicted in *B. melitensis* 16M and *B. abortus* 2308 (20, 21, 31) and very recently also in *B. canis* RM6/66 (32). Several upregulated or downregulated genes at the transcription level were found in the corresponding Δ*mucR* mutants, but apart from some flagellar proteins in *B. melitensis* 16M (21), no reports exist regarding whether the modification of the transcription rate of affected genes effectively leads to increased or reduced levels of the encoded proteins. In this work, we demonstrate that deletion of *mucR* in *B. ovis* PA leads to increased levels of transcripts for *hdeA, omp25d*, and *BOV_A0299* (Figure 5), resulting in increased translation of their respective encoded proteins, which were easily detected after SDS-PAGE, followed by Coomassie blue staining (Figure 1).

HdeA has been described in enteric bacteria as a periplasmic chaperone with activity at low pH and required for acid resistance (38, 39), and its ortholog in *B. abortus* 2308 was found to be involved in survival to *in vitro* acid stress exposure but dispensable for virulence in macrophages and mice (23). Considering its high overproduction in the Δ*mucR* mutant of *B. ovis* PA and its described role in resistance to acid pH, a condition encountered by *Brucella* inside phagocytes, we considered it interesting to analyze the relevance of HdeA for the *in vitro* and *in vivo* behavior of *B. ovis* PA and the isogenic Δ*mucR* mutant. However, no differences between the parental strain and the *B. ovis* PA Δ*hdeA* mutant were found in any of the tests performed, including survival at acid pH in the conditions assayed (Supplementary Table S1) and virulence (Figure 6). Accordingly, HdeA is not essential, at least in *B. abortus* 2308 and *B. ovis* PA, to survive under the acidic conditions that *Brucella* encounters inside phagocytes or to follow a normal infectious process in mice inoculated intraperitoneally. Nevertheless, HdeA of pathogenic enteric bacteria has exclusive activity at stomach pH ranges (pH values below 3)—conditions that have not been analyzed with *Brucella* Δ*hdeA* mutants—and is essential for bacterial resistance to this acidic environment (38, 39). Accordingly, although the intestinal mucosa is not considered a relevant port of entry for *Brucella* (40), HdeA could have a role if some degree of invasion by this route occurs. Moreover, contribution of HdeA to *Brucella* virulence in the natural host cannot be discarded. Although other characteristics not evaluated in this work could be affected, overproduction of HdeA in the *B. ovis* PA Δ*mucR* mutant (Figure 1) was not responsible for any of its observed defective characteristics (*i.e*., growth, OM-related properties, and virulence) nor provided beneficial characteristics to the Δ*mucR* mutant since the behavior of the Δ*mucR*Δ*hdeA* double mutant was undistinguishable from that of the Δ*mucR* single mutant in all tests performed.

Omp25d is a member of the Omp25/Omp31 family, which is constituted by seven homologous outer membrane proteins with a different occurrence and distribution pattern depending on the *Brucella* species (18). Detection of Omp25d in wild-type brucellae has not been reported, and this protein could only be detected in a *B. ovis* Δ*omp25d* mutant complemented *in trans* with *omp25d* and that overexpresses *omp25d* (18). The protein has been linked to the virulence of *B. ovis* PA (26), and its overproduction could be somehow involved in the attenuation of the Δ*mucR* mutant (Figure 6). In this respect, proteins of the Omp25/Omp31 family seem to be finely tuned (18), as it is also suggested by the lower transcription levels of *omp31* and *omp25c*—which encode two major OMPs of the family (18)—that were observed in the Omp25d-overproducing Δ*mucR* mutant (Figure 5) and that also seem to correlate with lower protein levels (Figure 4). According to these results, which include the lack of detection of Omp25d in *B. ovis* PA under standard culture conditions (Figures 1, 4A), MucR could modulate the levels of surface proteins of the Omp25/Omp31 family in response to environmental stimuli within the host to build an optimal surface architecture for the establishment of infection.

Regarding BOV_A0299, the third overproduced protein detected in the Δ*mucR* mutant of *B. ovis* PA (Figure 1), no homology with proteins of known function has been evidenced, but the protein is highly conserved in the genus *Brucella*, at least in the classical species (data not shown). According to the attenuation of the Δ*mucR* mutant and the degree of conservation of BOV_A0299 in the genus *Brucella*, this protein emerges as a new candidate to be further studied regarding its role in the biology of the bacterium.

The DNA region located upstream of *babR* (or *blxR*), a gene that is upregulated in the four available *Brucella* Δ*mucR* mutants (Table 4), has been previously analyzed in *B. abortus* 2308 while searching for the target site for MucR binding (33). Multiple binding sites of MucR to the promoter region of *babR* were identified, and it was determined that AT-rich regions containing T-A steps were involved in the MucR–promoter interaction resulting in transcriptional repression (33). Considering these observations, we have analyzed the DNA region located upstream of the genes coding for the three proteins overproduced in the *B. ovis* PA Δ*mucR* mutant (HdeA, Omp25d, and BOV_A0299). The three genes presented AT-rich regions containing T-A steps (Supplementary Figure S1) that could be targets for MucR binding, which suggests that MucR also acts as a direct repressor of these genes.

Despite the homology at the DNA level shared by the classical *Brucella* species (3), the Δ*mucR* available mutants show not only common transcriptomic characteristics but also differences in the expression of several genes (Table 4). Among the first genes that were shown to be regulated by MucR, several flagellar genes of *B. melitensis* 16M locus I (*fliF, fliC, ftcR*, and *flgE*) and that are required for virulence (41) can be mentioned (21). It was proposed that the downregulation of flagellar genes mediated by MucR was due to its repressor activity upstream of *ftcR*, which encodes a master regulator of flagellar expression (21). On the contrary, in *B. ovis* PA no MucR-mediated positive or negative regulation of *fliC* and *fliF* flagellar genes has been evidenced (Figure 5, Table 4). Nevertheless, this observation is not in disagreement with the proposed role of MucR as repressor of *ftcR* since the predicted binding site of FtcR upstream of *fliF* (42) is missing in *B. ovis* due to two independent deletion events accounting for 283 bp (11). Even if there was a MucR-mediated regulation of flagellar gene expression in *B. ovis* PA, deletion of *mucR* would not have a relevant impact in flagellum-dependent bacterial properties since it has been demonstrated that the entire three main flagellar loci are dispensable for *B. ovis* PA virulence, and their deletion does not modify any of the evaluated bacterial characteristics (15). Upregulation of flagellar genes detected in *B. canis* RM6/66 Δ*mucR* (32) was also in accordance with the results described for the *B. melitensis* 16M mutant (Table 4), but the expression of flagellar genes in *B. abortus* 2308 does not seem to be dependent on MucR (20) (Table 4). These differences cannot be explained according to distinctive traits in DNA regions located upstream of *fliC, fliF*, or *ftcR* since they are almost identical between *B. melitensis* 16M and *B. abortus* 2308 (data not shown). However, in addition to MucR and FtcR, other actors affecting flagellar gene expression have been identified in *B. melitensis* 16M (*e.g*., VjbR, BlxR, RpoE1, RpoH2, BdpA, and YbeY), which suggest a complex regulation network for flagellar gene expression (21). Therefore, the differences in flagellar gene expression detected among *Brucella* species in Δ*mucR* mutants could be related to different effects on the expression of the other regulators of the network, although the influence of the experimental conditions used in each work cannot be discarded.

Another recognized virulence factor in *Brucella*, including *B. ovis* PA, is the type IV secretion system whose components are encoded by the *virB* operon (11, 30). Although binding of MucR upstream of *virB1* has been evidenced in *B. abortus* 2308 (33), this interaction had a weaker affinity than that observed with the *babR* promoter and was considered to have little impact on the expression of *virB* genes in this strain (33). These observations corroborate previous results where no *virB* genes were listed among the differentially expressed genes in the Δ*mucR* mutant of *B. abortus* 2308 (20) and are in accordance with results obtained with the *B. canis* RM6/66 mutant. On the contrary, several *virB* genes were downregulated in the Δ*mucR* mutant of *B. melitensis* 16M (31), and reduced *virB2* transcription was detected in the *B. ovis* PA Δ*mucR* mutant (Figure 5, Table 4). Considering the low affinity of MucR by the *virB* promoter (33) and that DNA regions located upstream of *virB1* and *virB2* are almost identical in the four species (data not shown), differences among species regarding the role of MucR in *virB* expression are probably not related to a direct binding of MucR to the promoter region but to an indirect effect on other known or unknown regulators of this operon that is controlled by a complex regulatory network of repressors and activators responding to different environmental stimuli (30). BabR and VjbR, listed in Table 4, are two quorum-sensing-related transcriptional regulators known to affect *virB* expression, but several other proteins have been involved in its regulation (30). In this respect, the reported differences among *Brucella* strains regarding the production of VirB proteins under several culture conditions provide evidence of a differential regulation of *virB* expression within the genus *Brucella* (43).

The differences among *Brucella* spp. Δ*mucR* mutants were not exclusive to the transcriptomic pattern but also extended to phenotypic characteristics. A surprising observation was that, while the deletion of *mucR* caused *in vitro* growth defects in *B. melitensis* 16M (21), *B. abortus* 2308 (20), and *B. ovis* PA (Figure 2)—although they were less evident in *B. melitensis* 16M (21, 31)—the growth characteristics of the Δ*mucR* mutant of *B. canis* RM6/66 were identical to those of the parental strain (32). Also remarkable is the fact that, contrary to what was described in smooth *Brucella* Δ*mucR* mutants, *B. ovis* PA Δ*mucR* or Δ*mucR*Δ*hdeA* did not show higher susceptibility than the parental strain to oxidative, acid, or hypersaline stresses in the conditions assayed (Table 3, Supplementary Table S1). Additionally, *B. ovis* PA Δ*mucR* did not show the increased susceptibility to iron restriction (Table 3) reported for mutants of the three other *Brucella* species (20, 31, 32) despite the downregulation observed by the RT-qPCR of genes *ftrA* and *bfr* that are related to iron homeostasis (Figure 5). Susceptibility to detergents and the cationic peptide polymyxin B, which is related to properties of the bacterial cell envelope (5, 19), has been evaluated in the *Brucella* spp. Δ*mucR* mutants, except that of *B. canis*. While susceptibility to detergents is shared by the *Brucella* spp. Δ*mucR* mutants (20, 21) (Table 3), deletion of *mucR* increases the susceptibility of *B. melitensis* 16M and *B. ovis* PA (21) (Table 3), but not that of *B. abortus* 2308 (20) to polymyxin B. Considering the influence of MucR in the modulation of the bacterial surface architecture (clearly exemplified in this work by the increased levels of Omp25d in the *B. ovis* PA Δ*mucR* mutant), the differences observed among the *Brucella* spp. Δ*mucR* mutants might be related, at least in part, to the distinctive OM-related properties reported for each *Brucella* species (5, 19) and contribute to the differences of pathogenicity, host preference and tissue tropism that exist within the genus *Brucella*. Some of these distinctive properties seem to be associated to the smooth or rough phenotype, but some others seem to be dependent on differences in other cell envelope components (19). In this respect, a distinctive pattern for each *Brucella* species regarding members of the Omp25/Omp31 family, at least Omp25d being controlled by MucR (Figure 1), has been described (5, 18).

Despite the differences detailed above, all *Brucella* spp. Δ*mucR* mutants shared the virulence attenuation in cellular models and mice as a common trait (20–22, 32) (Figure 6). Although the *B. ovis* PA Δ*mucR* and Δ*mucR*Δ*hdeA* mutants have important *in vitro* growth defects (Figure 2), which would be a drawback for their industrial production, the persistent infection that they establish in mice (Figure 6) is an interesting trait that makes them potential candidates to be evaluated as specific attenuated vaccines against ovine brucellosis caused by *B. ovis*. The good protective activity against *B. melitensis* infection described for *B. melitensis* 16M Δ*mucR* (44) also encourages further evaluation of the *B. ovis* PA mutant as vaccine.

## Data Availability Statement

The raw data supporting the conclusions of this article will be made available by the authors, without undue reservation.

## Ethics Statement

The animal study was reviewed and approved by Bioethics Committee of the University of Salamanca and competent authority of Junta de Castilla y León, Spain.

## Author Contributions

RS-M and NV conceived the study. BT-C and NV wrote the manuscript, and all authors participated in the experimental work, the discussion of the results, and the revision of the manuscript. All authors read and approved the final version of the manuscript.

## Funding

This work is part of projects AGL2014-58795-C4-4-R, co-financed with FEDER funds, and PID2019-107601RB-C33, financed by MCIN/AEI/10.13039/501100011033. Financial support to BT-C was provided by project PID2019-107601RB-C33, financed by MCIN/AEI/10.13039/501100011033. The projects were awarded by Ministerio de Ciencia e Innovación, Spain.

## Conflict of Interest

The authors declare that the research was conducted in the absence of any commercial or financial relationships that could be construed as a potential conflict of interest.

## Publisher's Note

All claims expressed in this article are solely those of the authors and do not necessarily represent those of their affiliated organizations, or those of the publisher, the editors and the reviewers. Any product that may be evaluated in this article, or claim that may be made by its manufacturer, is not guaranteed or endorsed by the publisher.
