# Supplementary material for: The Transcriptional Regulator MucR, but Not Its Controlled Acid-Activated Chaperone HdeA, Is Essential for Virulence and Modulates Surface Architecture and Properties in Brucella ovis PA
Source: Front Vet Sci. 2022 Jan 31;8:814752. doi: 10.3389/fvets.2021.814752 (PMC8843074; doi:10.3389/fvets.2021.814752)
Supplement: Supplementary file 1 [file Data_Sheet_1.PDF]

Supplementary Figure 1. *B. ovis* 63/290 intergenic regions upstream *babR* **(A)**, where MucR is known to bind in *B. abortus* 2308 (1), and genes encoding HdeA **(B)**, Omp25d **(C)** and BOV_A0299 **(D)**, the three proteins highly overproduced in *B. ovis* PA **(Figure 1).** The beginning of open reading frames for each gene are highlighted in blue. In *babR* **(A)**, regions known to interact with MucR are underlined, and T-A steps required for optimal binding are highlighted in red (1). In the other genes, AT-rich regions are underlined and T-A steps that could be involved in interaction with MucR are highlighted in red.

(A) ***babR* (*blxR*)**

ATTTTCATTATCT**AATTTAGAATGAAGTTATATTCAATATAAAAGTAGAATTTTGAAAATCGCTAA**
TTATGATGAAAGTAATCTTCGTAGAAGAAATATGAAAGATATGCTAGGAATGTTTAGCGATACATTT
TACCAAAAATCGCTGCGATTCCAAGAATTCATATTTATTTTGAAACGCTACTGGG **ATG TCT GCA**
ATG AAA TGG

(B) ***hdeA***

GGAAGGTCTCGGGATGATATTAATATACATAGTGAAACTTGAAGTGATTATATAAAAATTATGTATA
TTAGAGGATTGTCATGGACAATCCTTGTAGGATAATATAGAAATGAGACGTGTTACTATATCTAAGC
GAAGGAGAAGGAA **ATG ATT AAG ACT CTC TTC**

(C) ***omp25d***

TTTTTTTATGTCAATCCTCACCCAATATTTGATATTCAATTTAAATATTAGATATATATGAAGTATTAC
TGCATTAAATTTCAAGTTAATCAAATATATATTAACTTTTTGTATAAATGAAAATCAAGACAATTCAT
TATTGTTGCTCATTTACTACATACATTCGACCAGATCATCACTATATGCTAGCCGCATTGCAATGCCA
TTCTTATTAGGAGTTTTCA **ATG ACG TTC AAA AAT CTA**

(D) ***BOV_A0299***

TCACAGCATATATCCATGAATATTTTTAAGTTATTTCTCAATAACAACGCGCTAAATTCCGTTGTTTTTT
CATGGTTTCTGCATGAATTATCAATTCATAACATTGCCCGCAAAACAAGTAATCAAAAAATTCGTTGTG
AATCTTGCCGCCATAAAATTATACTCTATTTATAGTGACTACAGATCCCGACGCGATAGTCACGAATCC
ATCGGCAGTCGGGGGCATAATTTTAACAACTGGAGTTTGAC **ATG GGC ATT TTC GAC AAG**

REFERENCE

(1) Borriello G, Russo V, Paradiso R, Riccardi MG, Criscuolo D, Verde G, et al. Different Impacts of MucR Binding to the *babR* and *virB* Promoters on Gene Expression in *Brucella abortus* 2308. *Biomolecules*. (2020) 10. 10.3390/biom10050788
