# Supplementary material for: The Transcriptional Regulator MucR, but Not Its Controlled Acid-Activated Chaperone HdeA, Is Essential for Virulence and Modulates Surface Architecture and Properties in Brucella ovis PA
Source: Front Vet Sci. 2022 Jan 31;8:814752. doi: 10.3389/fvets.2021.814752 (PMC8843074; doi:10.3389/fvets.2021.814752)
Supplement: Supplementary file 2 [file Table_1.pdf]

Supplementary TABLE 1 | Susceptibility of the *B. ovis* PA mutants to acid and hypersaline stress [a].

	Log_{10} CFU/ml (mean ± SD)	
**Strain**	**pH 4,4**	**1M NaCl**
***B. ovis* PA**	7.84 ± 0.24	6.91 ± 0.08
**Δ*mucR***	7.74 ± 0.20	6.73 ± 0.28
**Δ*mucR* comp**	7.78 ± 0.18	7.00 ± 0.08
**Δ*mucR*Δ*hdeA***	7.11 ± 0.46	6.83 ± 0.14
**Δ*hdeA***	7.86 ± 0.08	6.90 ± 0.09

[a] Assays were performed on TSB-YE-HS adjusted to pH 4.4 or containing 1M NaCl as described in Materials and Methods. Bacterial counts were determined after exposure for 24 h. No statistically significant differences were observed between the mutants and the parental strain *B. ovis* PA (P >0.01).
